# A Wearable Ballistocardiography Device for Estimating Heart Rate During Positive Airway Pressure Therapy: Investigational Study Among the General Population

**DOI:** 10.2196/26259

**Published:** 2021-05-05

**Authors:** Mark Gardner, Sharmil Randhawa, Gordon Malouf, Karen Reynolds

**Affiliations:** 1 Medical Device Research Institute Flinders University Clovelly Park Australia; 2 ACRF Image X Institute Eveleigh Australia; 3 ResMed Asia Operations Ptd Ltd Singapore Singapore

**Keywords:** heart rate, ballistocardiography, sleep apnea, positive airway pressure, gyroscope, Kalman filter

## Abstract

**Background:**

Obstructive sleep apnea (OSA) is a condition in which a person’s airway is obstructed during sleep, thus disturbing their sleep. People with OSA are at a higher risk of developing heart problems. OSA is commonly treated with a positive airway pressure (PAP) therapy device, which is used during sleep. The PAP therapy setup provides a good opportunity to monitor the heart health of people with OSA, but no simple, low-cost method is available for the PAP therapy device to monitor heart rate (HR).

**Objective:**

This study aims to develop a simple, low-cost device to monitor the HR of people with OSA during PAP therapy. This device was then tested on a small group of participants to investigate the feasibility of the device.

**Methods:**

A low-cost and simple device to monitor HR was created by attaching a gyroscope to a PAP mask, thus integrating HR monitoring into PAP therapy. The gyroscope signals were then analyzed to detect heartbeats, and a Kalman filter was used to produce a more accurate and consistent HR signal. In this study, 19 participants wore the modified PAP mask while the mask was connected to a PAP device. Participants lay in 3 common sleeping positions and then underwent 2 different PAP therapy modes to determine if these affected the accuracy of the HR estimation.

**Results:**

Before the PAP device was turned on, the median HR error was <5 beats per minute, although the HR estimation error increased when participants lay on their side compared with when participants lay on their back. Using the different PAP therapy modes did not significantly increase the HR error.

**Conclusions:**

These results show that monitoring HR from gyroscope signals in a PAP mask is possible during PAP therapy for different sleeping positions and PAP therapy modes, suggesting that long-term HR monitoring of OSA during PAP therapy may be possible.

## Introduction

### Background

Obstructive sleep apnea (OSA) is a condition in which a person’s upper airway is obstructed during sleep [1]. This leads to disrupted breathing, which affects the sleep quality. It affects 14% of men and 5% of women aged between 30 and 70 years [2]. In addition to having reduced sleep time and quality, people with OSA are at a risk of developing heart problems [3]. People with heart problems are also advised to be checked for OSA [4]. OSA is commonly treated using positive airway pressure (PAP) therapy, in which a device is used to keep the airway from becoming obstructed by the application of a positive pressure [1].

It is thought that by long-term monitoring of the heart rate (HR) of people with OSA, it may be possible to monitor changes in heart health. If individual heartbeats can be accurately detected, then the HR variability of the wearer can be used to estimate cardiac health or predict heart problems or people undergoing PAP therapy [5]. However, if HR variability measurements are not possible, measuring the resting HR [6,7] can also be used as a predictor of heart failure. Finally, OSA episodes are characterized by acute changes in HR [8]; thus, by monitoring HR, OSA episodes could be detected [9], which could be used to help evaluate the effectiveness of PAP therapy in situations where there is significant mask leakage.

Adding HR monitoring to PAP therapy offers a good opportunity for long-term continuous cardiac monitoring as, when used correctly, PAP therapy is used for several hours every night. To add HR monitoring to PAP therapy, it would be advantageous to integrate sensors into the PAP mask rather than adding additional devices to the PAP therapy setup. However, any sensors or devices that are embedded into a PAP mask must be comfortable, safe, and noninvasive to promote patient compliance. In addition, as it is recommended that PAP therapy masks be replaced regularly to prevent air leakage, any modifications to the PAP masks should be low cost. This will ensure that there is no significant increase in the cost of the masks, leading to a more expensive PAP therapy. A more detailed description of the case for a low-cost device for monitoring HR during PAP therapy can be found in the thesis by Gardner [10].

We previously proposed a device consisting of a modified PAP mask that simultaneously measures electrocardiogram (ECG) and photoplethysmography (PPG) signals from the wearer [11]. However, both ECG signals [12] and PPG signals [13] can be affected by motion artifacts, which during PAP therapy can occur from whole body movements that occur naturally during sleep. Motion can be detected using an accelerometer or a gyroscope to exclude the signal affected by motion artifacts, as described by He et al [14]. However, costs can be reduced by using only one sensor on the mask instead of using multiple sensors. Hence, if the HR of the wearer can be detected from the sensor used to detect movement, then only one sensor is needed.

Another advantage of using a movement sensor to detect HR instead of ECG or PPG is that more variables beyond HR can be extracted. The gyroscope and accelerometer signals used to measure ballistocardiography (BCG) also have the potential to measure variables such as respiration and sleep position, as well as detecting movement during sleep [15]. Indeed, we have previously shown that significant head movement can be detected by monitoring the magnitude of a gyroscope signal mounted on a PAP mask [16].

BCG (also known as seismocardiography) is a method for detecting HR by detecting small movements or vibrations caused by heartbeats [17]. BCG-based devices integrated into beds have been shown to be able to monitor HR during sleep and detect apnea episodes [18,19]. Wearable BCG devices have been developed for cardiac monitoring, in which an accelerometer or a gyroscope is positioned such that it rests on the patient’s skin [14,20-24]. Most wearable BCG devices involve the sensor being placed on the wearer’s chest, as this is the optimum location for cardiac monitoring [20-23]. However, if the sensors need to be integrated into the mask for PAP therapy monitoring during PAP therapy, the sensors cannot be placed on the chest.

Previous head-mounted BCG devices for HR monitoring have been reported in the literature. Hernandez et al [24] used the signals from the on-board inertial measurement unit (IMU) in Google Glass, a wearable headset in the shape of glasses. The HR and respiration rate were estimated from the accelerometer and gyroscope signals from the IMU. This device was able to estimate the HR most accurately when the participants were lying on their back compared with standing and sitting, supporting the concept of using a similar technique for monitoring during sleep.

Floris et al [25] conducted a similar study in which HR and respiration rate were estimated from signals from an accelerometer and a gyroscope mounted inside a head-worn virtual reality device. Similar to the study by Hernandez et al [24], in the study by Floris et al [25], participants wore the device while standing, sitting, and lying down, and the HR was estimated over sliding 10-second windows. However, unlike the results by Hernandez et al [24], the results presented by Floris et al [25] showed more accurate HR estimation when the participants were standing up compared with when the participants were lying down.

He et al [14] developed a wearable BCG device mounted behind the ear, which contained an accelerometer and ECG electrodes. Heartbeat information was extracted from the accelerometer signals. However, unlike the device by Hernandez et al [24], which measured HR, He et al [14] measured the time delay between the accelerometer signal and the on-board ECG signal, using it to estimate the pre-ejection period in the cardiac cycle. This value was estimated over an 8-second period, owing to the relatively poor signal-to-noise ratio (SNR) of both the measured ECG and BCG signals. In addition, He et al [14] found that for 7 healthy subjects, the amplitude of the accelerometer signal correlated with the stroke volume of the wearer (*R*^2^=0.66).

Most BCG examples that have been previously developed have a gyroscope or an accelerometer placed on the person’s chest. Floris et al [25] described how, compared with these chest BCG signals, BCG signals measured from the head or neck have a lower SNR and are more prone to motion artifacts. In the examples of head-worn BCG devices, the authors compensate for this low SNR by taking an average HR over a period of either 8 [14], 10 [25], or 20 seconds [24] instead of measuring an instantaneous HR. A similar result was also shown for a BCG wearable device located on the wrist [26]. This low SNR makes the accurate monitoring of HR from more proximal locations, such as the head, more difficult than monitoring from the chest.

We have previously reported a device consisting of a gyroscope attached to a PAP mask and a method for extracting HR information from the gyroscope signals [16,27]. The advantage of this device is that it is integrated into the PAP device setup, meaning that no extra devices need to be worn for the wearer to have their HR monitored. It is also a low-cost and simple method for monitoring the HR. Finally, in comparison with other wearable BCG devices located on the head, the device proposed in this study has been shown to provide an accurate HR value every 1.5 seconds, as opposed to averaging an HR value over a period of several seconds. However, the device was only tested on one participant, with no indication of interpatient variability.

### Objectives

This study aims to evaluate the accuracy of the proposed HR estimation method on a group of healthy participants, verifying that this concept works on a broader population. This is the first study to evaluate the accuracy of HR estimation using a BCG-based sensor mounted on a PAP mask on multiple participants. In addition, this is the first study to investigate the effect of different PAP therapy modes on the accuracy of the BCG-based HR estimation method.

## Methods

### Overview

The HR estimation process involved first collecting the BCG signal from the participants while they were wearing a continuous positive airway pressure (CPAP) mask. The signals were then retrospectively analyzed, and heartbeats were detected. From these detected heartbeats, a data fusion method was used to produce a consistent and accurate HR signal. The details of how each step of this process was achieved are described in this section.

### Experiment Setup

**Figure 1:**
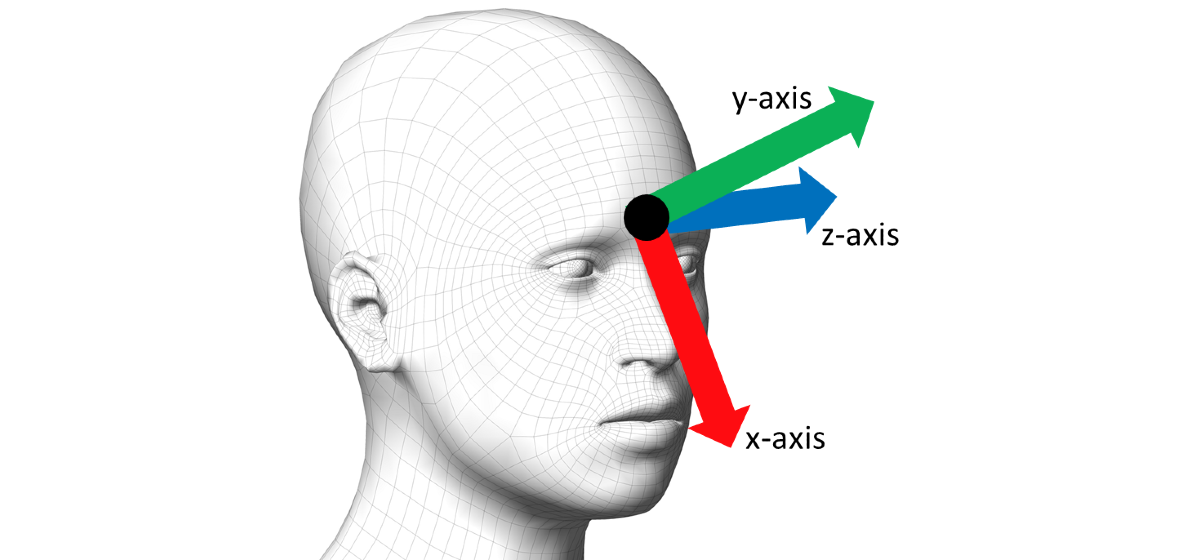
Position and orientation of the gyroscope on a positive airway pressure mask.

A PAP mask was modified to estimate the HR of the wearer. The mask was a ResMed Quattro Air mask, onto which an IMU (MPU 9150; Invensense), which includes a 3-axes gyroscope signal, was attached, as shown in Figure 1. The configuration of the gyroscope was such that x rotation corresponded to rotating the head from left to right, y rotation corresponded to head tilt toward the shoulders, and z rotation corresponded to a nodding up and down movement.

The gyroscope was connected to an Arduino Pro Mini, and the signals were collected at a sampling frequency of 50 Hz. All experimental signals were recorded using Labview (National Instruments) and were analyzed post experiment using MATLAB (Mathworks).

The participants were also connected to a PAP device (ResMed Lumis 150, ResMed) during recording to simulate PAP therapy.

The device was tested on 19 participants (14 males and 5 females), with a mean age of 30 (SD 9) years. OSA was not an exclusion criterion for participation in this study. The experiment was approved by the Southern Adelaide Human Research Ethics Committee.

**Figure 2:**
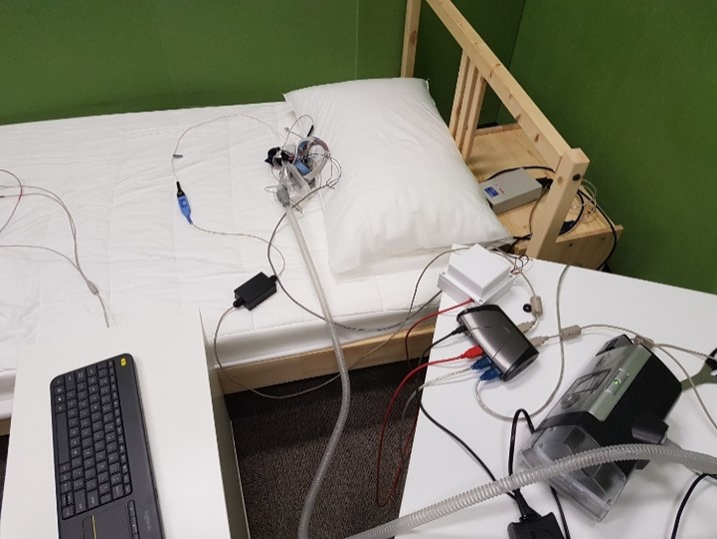
The experiment setup. The participant will wear the modified positive airway pressure (PAP) mask and lie on the bed in the required orientation. The PAP mask was connected to the PAP device as the gyroscope signals were recorded.

The participants lay on a bed, lying on their back, left, and right side for a period of 5 minutes in each position (stages 1-3; Table 1). When the participants were lying on their side, they were instructed to lie on their side in a way that was comfortable for them and similar to how they would lie when sleeping. This was done to determine whether the sleeping position affected the accuracy of the HR measurement. The PAP device was turned off during the first 4 stages of the experiment. The experimental setup is shown in Figure 2.

**Table 1:** Participants’ positions and positive airway pressure therapy modes for the experiment. Each experiment stage lasted for 5 minutes.

Experiment stages	Participant position	PAP^a^ mode
1	Lying on the back	Off
2	Lying on the left side	Off
3	Lying on the right side	Off
4	Lying on the back	Off
5	Lying on the back	CPAP^b^
6	Lying on the back	VPAP^c^

^a^PAP: positive airway pressure.

^b^CPAP: continuous positive airway pressure.

^c^VPAP: variable positive airway pressure.

The participants then lay on their back (stage 4), and the PAP therapy device was turned on, using 2 different PAP modes (stages 5 and 6; Table 1). These modes were CPAP with a pressure of 6 cm H_2_O and variable positive airway pressure (VPAP) with pressures of 4 cm H_2_O during expiration and 8 cm H_2_O during inspiration. The applied pressures were in the lower range of clinical PAP pressures [28], so that the participants would not feel too uncomfortable.

A heartbeat detected from the gyroscope signal was determined as correctly detected if it was within 0.02 seconds of a heartbeat detected in the reference ECG signal [29]. The HR values estimated from the gyroscope signal (methods described below) were compared with HR values from a reference ECG signal, measured using 3 Ag/AgCl electrodes (Red Dot electrodes, 3M) placed on the participant’s hands and right foot. The ECG heartbeats were detected using the Pan-Tompkins heartbeat detection algorithm [29].

As there is a natural delay between the timing of the heartbeat in the ECG and the gyroscope signals [14,30], the heartbeats detected from the gyroscope signals were shifted back in time to compensate for this delay. The value of this delay was calculated using the median time difference between the detected heartbeats in the ECG signal and the gyroscope signals.

The heartbeat detection sensitivity and false positive rate (FPR) were calculated:

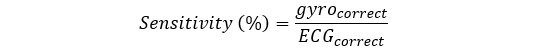

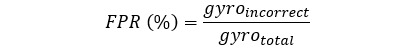





where *ECG_correct_* and *gyro_correct_* are the number of correctly detected heartbeats in the ECG and gyroscope signals, respectively; *gyro_incorrect_* is the number of heartbeats not associated with a heartbeat from an ECG signal, and *gyro_total_* is the total number of heartbeats detected in the gyroscope signal.

### Heartbeat Detection Algorithm

**Figure 3:**
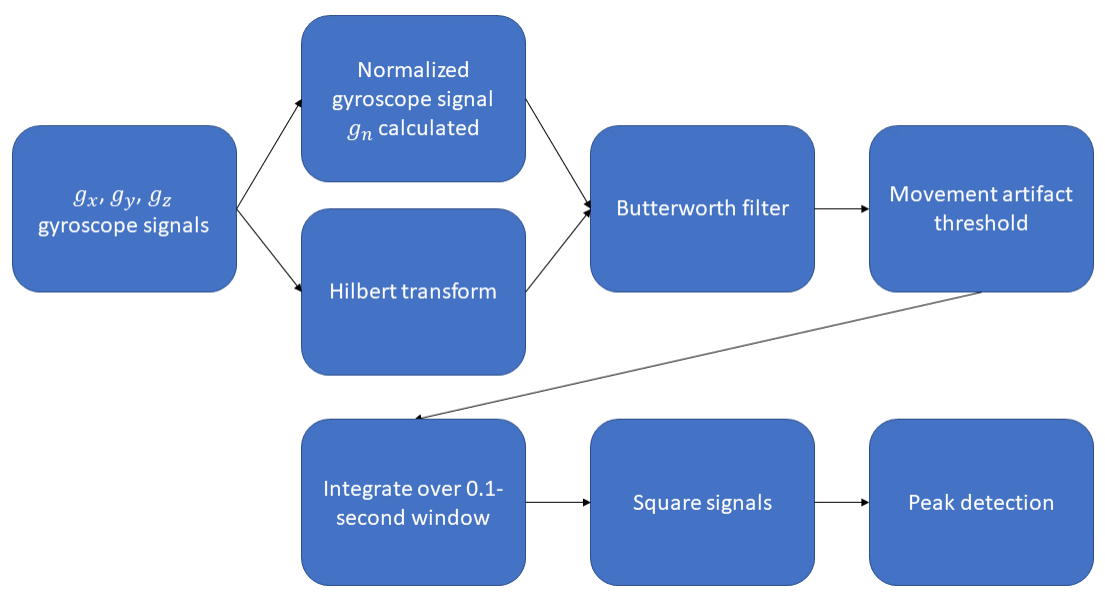
A summary of the proposed method for transforming the gyroscope signal to enable heartbeat detection.

The method for identifying heartbeats in a gyroscope signal mounted on a PAP therapy mask has been described previously and is summarized in Figure 3 [27]. Briefly, a normalized gyroscope signal (*g_n_*) was derived using the x, y, and z gyroscope signals *(g_x_, g_y_, g_z_)* such that:

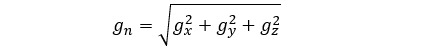



All signals were resampled to 500 Hz, similar to the study by Hernandez et al [26]. The signals *g_x_, g_y_, g_z_* and *g_n_* were then transformed to maximize the SNR using the methods previously described [27]. A movement threshold was also created, such that when the signal magnitude exceeded this threshold, heartbeat detection was paused, as movement artifacts significantly reduced the accuracy of the heartbeat detection algorithm [16].

For the heartbeats detected in the *g_x_, g_y_, g_z_* and *g_n_* signals, the sensitivity and FPR were calculated and compared between the different experiment stages.

### Kalman Filter for Data Fusion

A data fusion method was developed to combine the HR information from the gyroscope signals *g_x_, g_y_, g_z_* and *g_n_*. This has been described and implemented on one subject in our previous work [16,27]. A Kalman filter (KF) is a data fusion method that is commonly used in fields such as robotics [31], but it has also been used in physiological monitoring to produce accurate and consistent HR measurements [32]. We have previously shown that the KF algorithm described has superior performance when compared with a simple moving average [10,16,27].

To implement the KF algorithm, the recording period was first divided into nonoverlapping 1.5-second windows. The purpose of these windows is to create discrete and relatively large time intervals for the KF. A width of 1.5 seconds was chosen such that for a subject with a normal HR (>40 beats per minute [BPM]), there will be at least one heartbeat per window. Each window was analyzed such that one HR value was extracted per signal, and outlier HR values (less than 40 or greater than 200 BPM) were discarded.

The HR was modeled such that for time *k*:

*HR_k_* = *HR_k_*_−1_ + *ω_k_***   (1)**

where *w_k_* represents the natural variation of the HR, modeled as zero-mean Gaussian noise with covariance *Q_k_*. At time *k*, the observation measurements were defined as:

*z_k_* = *H_k_x_k_* + *v_k_***   (2)**

where:

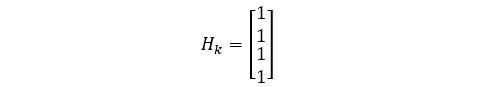

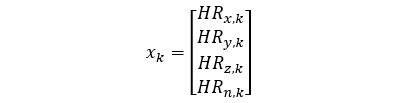





*HR_x,k_*, *HR_y,k_*, and *HR_z,k_* are the HR estimations from the x, y, and z components, respectively, at time *k*, and *HR_n,k_* is the HR estimation from the normalized gyroscope component. In addition, *R_k_* was defined as:

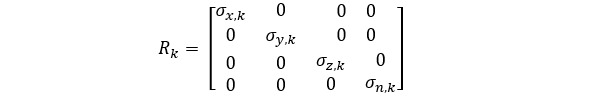



where the component *i* at time *k*:

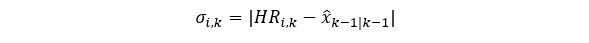



As the instantaneous HR signal from the ECG does not have a regular time interval between measurements, the ECG signal was resampled to be a fixed interval signal. Windows of width 1.5 seconds were created, similar to the KF method, and the ECG HR values inside each window were averaged to produce a ECG HR signal with a fixed interval of 1.5 seconds. The HR error for the KF was then defined as the magnitude difference between the KF output and the ECG HR for each 1.5-second window. The mean HR error was calculated and analyzed for each experiment stage.

## Results

### Heartbeat Detection Algorithm

**Figure 4:**
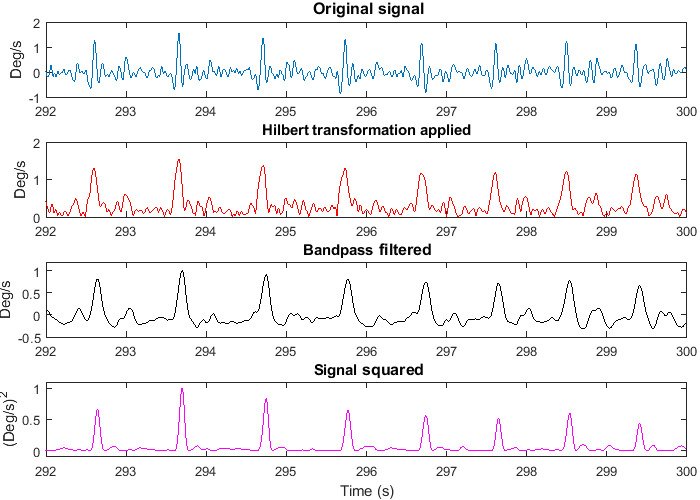
An example of how the raw gyroscope signal is transformed to a signal where peak detection can be easily applied. First, the raw signal (top) has a Hilbert transformation applied (second from top). This signal then has a bandpass filter applied (third from top). The signal is finally squared (bottom).

Figure 4 shows the output from the heartbeat detection algorithm applied to a raw gyroscope signal. Peaks due to the motion of the heartbeat are easily visible. The percentage of heartbeats that were correctly detected in each participant position by the individual and combined gyroscope signals is given in Table 2. Table 3 shows the percentages of false positives. From these tables, it can be seen that the heartbeat detection algorithm was most successful in detecting heartbeats in the Y gyroscope signal, which represents the lateral movement of the head toward the shoulders (Figure 1).

**Table 2:** Median (IQR) percentage of heartbeats that were correctly identified by the heartbeat detection algorithm.

Experiment stage^a^	X gyroscope, median (IQR)	Y gyroscope, median (IQR)	Z gyroscope, median (IQR)	Normalized gyroscope, median (IQR)
Lying on the back	83.84 (28.02)^b^	94.28 (18.17)	72.88 (22.53)^b^	92.21 (15.13)
Lying on the left side	52.61 (20.80)^b,c^	71.80 (33.11)^c^	56.23 (19.56)^c^	62.64 (32.01)^c^
Lying on the right side	59.44 (26.32)^b,c^	76.82 (28.56)^c^	55.54 (19.21)^b,c^	68.83 (18.23)^c^
Lying on the back	81.79 (25.97)^b^	90.65 (12.76)	66.19 (19.64)^b^	89.39 (25.36)
CPAP^d^ on	84.79 (25.06) (*P*=.06)	90.05 (17.76)	65.79 (22.94)^b^	90.69 (19.76)
VPAP^e^ on	77.78 (26.25) (*P*=.05)	90.96 (16.18)	39.74 (26.17)^b,c^	59.67 (33.11)^b,c^

^a^Significance calculated using paired sign tests due to nonnormal distributions.

^b^Percentage of heartbeats detected significantly less than detected in the Y gyroscope signal (*P*≤.037).

^c^Decrease in median heartbeats detected compared with lying on the back (*P*≤.047).

^d^CPAP: continuous positive airway pressure.

^e^VPAP: variable positive airway pressure.

**Table 3:** Median (IQR) percentage of heartbeats that were incorrectly classified as heartbeats by the heartbeat detection algorithm.

Experiment stage^a^	X gyroscope, median (IQR)	Y gyroscope, median (IQR)	Z gyroscope, median (IQR)	Normalized gyroscope, median (IQR)
Lying on the back	12.51 (37.22)^b^	4.34 (7.10)	22.09 (25.08)^b^	5.91 (9.85)
Lying on the left side	42.42 (24.34)^b,c^	16.63 (22.78)^c^	33.51 (25.68)^c^	30.95 (32.63)^c^
Lying on the right side	29.98 (14.78)^b,c^	8.55 (17.46)^c^	31.05 (18.95)^b,c^	22.09 (16.97)^c^
Lying on the back	11.17 (31.19)^b^	5.23 (11.55)	25.66 (25.13)^b^	6.81 (10.63)
CPAP^d^ on	14.88 (17.66)	4.61 (12.33)	25.48 (21.10)^b^	6.23 (10.98)
VPAP^e^ on	17.86 (25.24)	8.07 (13.79)	37.25 (22.46)^b,c^	15.62 (13.36)^b,c^

^a^Significance calculated using paired sign tests due to nonnormal distributions.

^b^Percentage of detected heartbeats significantly greater than detected in the Y gyroscope signal (*P*≤.024).

^c^Decrease in median heartbeats detected compared with lying on the back (*P*≤.038).

^d^CPAP: continuous positive airway pressure.

^e^VPAP: variable positive airway pressure.

Tables 2 and 3 also show that the Z gyroscope signal (corresponding to forward head tilt) produced the lowest percentage of correct heartbeats and the highest proportion of false heartbeats for all sleeping positions.

Tables 2 and 3 show that when the participants were lying on either side, the heartbeat detection method was significantly less effective than when the participants were lying on their back.

**Figure 5:**
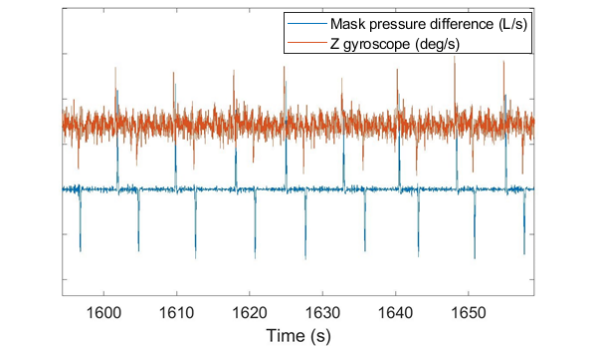
An example of how the change in pressure during variable positive airway pressure (blue) causes artifacts in the gyroscope signal (red).

The results also showed that there was no difference between the effectiveness of the heartbeat detection accuracy when the PAP device was off (stage 4) and when the CPAP mode was on (stage 5). However, the rapid change in pressure that occurs during the VPAP therapy mode (stage 6) created motion artifacts in the Z gyroscope signal, leading to an increase in the FPR and a reduction in the sensitivity. An example of these motion artifacts caused by the rapidly changing pressure is shown in Figure 5.

In summary, the results in Tables 2 and 3 show that accurate heartbeat detection is possible when the participants are lying on their back, particularly for the Y gyroscope signal. However, the heartbeat detection algorithm was less effective when the participants were lying on the side. The heartbeat detection effectiveness was also reduced when the VPAP therapy mode was activated.

Although the heartbeat detection algorithm shows promising results, the results are not consistent enough for continuous accurate HR monitoring, particularly when the participants were lying on their side. Similar results were observed in previous preliminary testing [16,27]. Given that from the gyroscope 4 signals were recorded that all measured HR, the next step was to implement a data fusion method to investigate whether this would enable a consistent and accurate HR measurement from the BCG signals.

### KF for Data Fusion

**Figure 6:**
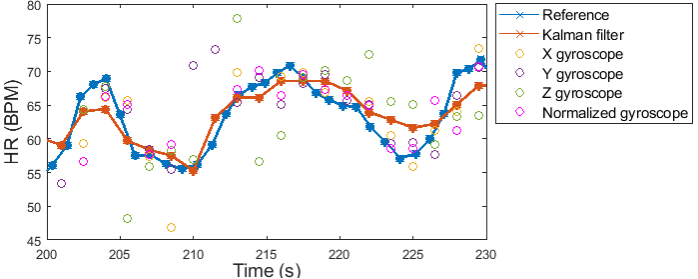
An example of how the heart rate (HR) information from the gyroscope signals are used to estimate the HR using the Kalman filter compared with the reference electrocardiogram HR. BPM: beats per minute; HR: heart rate.

An example of how the described KF algorithm can perform data fusion to estimate HR is shown in Figure 6. This figure shows good consistency between the output of the KF algorithm and the HR from the reference ECG signal, even when the HR values from the individual gyroscope components were less reliable.

**Figure 7:**
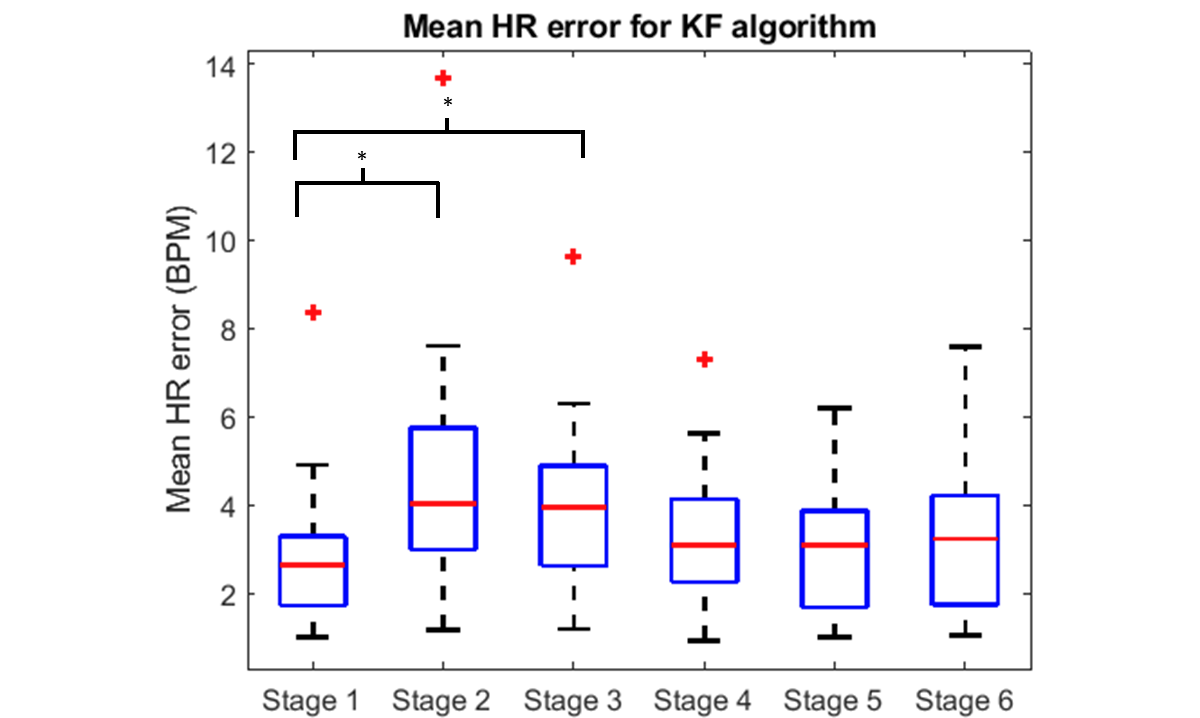
Mean error of the estimated heart rate from the Kalman filter. “*” indicates significant difference (*P*<.05). Significance was calculated using paired one-tailed *t* tests. BPM: beats per minute; HR: heart rate; KF: Kalman filter.

The mean HR error of all participants for all the experiment stages is shown in Figure 7. The accuracy of the HR estimation from the KF was reduced when the participants were lying on their side (stages 2-3) compared with when they were lying on their back in stage 1. This is shown in Figure 7 by the 1.5 BPM increase in the mean error when the participants were lying on their side (*P*≤.02). Figure 7 also showed that there was no significant difference in the mean error between when the participants were lying on their left or right side (*P*≥.32).

Figure 5 shows that the VPAP mode (stage 6) caused motion artifacts in the Z gyroscope signal, which reduced the effectiveness of the heartbeat detection algorithm, as shown in Tables 2 and 3. However, Figure 7 shows that the activation of the VPAP mode (stage 6) did not have a significant effect on the error of the KF HR estimation when compared with the CPAP mode (stage 5; *P*≥.14). Hence, the KF algorithm is able to compensate for the motion artifacts in the Z gyroscope signal, ensuring no change in accuracy during VPAP mode.

## Discussion

### Principal Findings

In this study, a low-cost modified PAP therapy mask was created to estimate the HR of the wearer using the signals from the on-board gyroscope. A heartbeat detection algorithm was developed to identify heartbeats in the gyroscope signals, and a KF algorithm was implemented in an attempt to provide a more consistent and accurate HR signal. The KF algorithms were tested with healthy participants lying on their back and sides and with participants simulating 2 different PAP therapy modes.

The results in Tables 2 and 3 suggest that the heartbeat signal is strongest in the Y gyroscope direction. Given the sensor orientation shown in Figure 1, this is an unexpected result. The results of this study suggest that the complex anatomical structures between the heart and the head cause the head to rotate the strongest in the Y gyroscope direction and impede the gyroscope signal in the Z direction. Future work could study the relationship between the heartbeat and head movement. Alternatively, other methods for detecting instantaneous HR from a BCG signal that have been previously developed [33], including machine learning–based heartbeat detection algorithms [34,35], may be able to increase the accuracy of heartbeat detection when the participants are lying on their side.

Few devices have been developed for monitoring HR during sleep using only a BCG signal. Di Rienzo et al [23] used a wearable device that contained an accelerometer located on the sternum of the wearer to monitor cardiac intervals during sleep. However, to monitor these intervals, the BCG signal information was combined with an ECG signal that was used to locate and identify the heartbeats. The results from this study show that a standalone BCG device can be used to estimate HR during sleep, although this does not have the same heartbeat detection accuracy as systems that use only the ECG signal [23] to detect the heartbeats.

Hernandez et al [26] developed a device to measure HR using only the BCG signals. The algorithm developed by Hernandez et al [26] was designed such that an HR could be accurately measured when the participant was standing, sitting, and using the device in normal everyday life. This meant that it needed to be much more resistant to movement artifacts than if it was designed for just sleep monitoring. As a result, Hernandez et al [26] did not collect beat-by-beat HR information and instead used the peak signal frequency over a 20-second interval as the HR value. This method for HR estimation was designed such that it would require low power and low computational cost.

In our study, a shorter time interval could be used to estimate the HR, as movement episodes are less likely. However, the trade-off between the decrease in the signal interval length is that there is a slight decrease in the accuracy of the HR estimation, although it is difficult to compare accuracies over different time intervals. Hernandez et al [26] were able to estimate the HR of the wearer to within 0.44 BPM of the reference HR value over a 20-second interval when the participants were lying on their back. In contrast, the mean error of the HR estimation from the KF algorithm when the participants were lying on their back was approximately 3 BPM. In addition to being able to track HR changes quicker than other examples in the literature, by integrating the sensors into the PAP mask, no additional devices are required to be worn by people using PAP devices.

The sensor used in this study (MPU-9150) was sampled at a sampling rate of 50 Hz and then up-sampled to 500 Hz. This is much lower than that of other BCG examples, which have significantly higher sampling rates [20]. A sensor with a frequency of 50 Hz was chosen to keep the cost of the device low, and the trade-off of reducing the cost is that the resolution and sampling rate are lower. The results of this study show that a sampling rate of 50 Hz is sufficient to estimate HR at 1.5-second intervals using the described algorithm.

### Limitations

This study had several limitations. The participants of this study were not limited to people with OSA or people with cardiac or respiratory problems. In addition, the participants were not sleeping during the study but were lying awake. Although the results of this study show that the proposed method works well on healthy participants, future work will look at the effectiveness of the proposed method on people with OSA who are sleeping.

The applied pressures that were used during the CPAP (6 cm H_2_O) and VPAP modes (4 cm H_2_O-8 cm H_2_O) were relatively low compared with the pressures used clinically for PAP therapy modes. These pressures were chosen to ensure the comfort of the participants, many of whom had not previously undergone PAP therapy. It is unknown whether for higher pressures, the results would change significantly.

The HR model used in the KF is a simplistic estimation of the HR dynamics during sleep. Given that it is possible to monitor additional variables using the gyroscope signals, it is possible to increase the HR estimation accuracy by increasing the complexity of the HR model. Future work will look at further developing the HR model used in the KF.

### Conclusions

In this study, the ability to accurately measure HR from a gyroscope attached to a PAP mask has been shown. The results show that our previously developed method for estimating HR was able to estimate HR accurately for healthy participants regardless of their sleeping position. In addition, the CPAP and VPAP therapy modes did not significantly affect the HR estimation accuracy, despite the change in pressure of the VPAP mode causing artifacts in the gyroscope signal. The results of this study suggest that long-term monitoring of the HR of a person using a PAP device is possible. Future testing will involve testing the device during sleep and in patients with sleep apnea during PAP therapy and investigation of the device’s response during arrhythmias.

## Abbreviations

BCG: ballistocardiography
BPM: beats per minute
CPAP: continuous positive airway pressure
ECG: electrocardiogram
FPR: false positive rate
HR: heart rate
IMU: inertial measurement unit
KF: Kalman filter
OSA: obstructive sleep apnea
PAP: positive airway pressure
PPG: photoplethysmography
SNR: signal-to-noise ratio
VPAP: variable positive airway pressure

## References

[1] Patel SR (2019). Obstructive sleep apnea. Ann Intern Med.

[2] Peppard PE, Young T, Barnet JH, Palta M, Hagen EW, Hla KM (2013). Increased prevalence of sleep-disordered breathing in adults. Am J Epidemiol.

[3] Mehra R, Benjamin EJ, Shahar E, Gottlieb DJ, Nawabit R, Kirchner HL, Sahadevan J, Redline S (2006). Association of nocturnal arrhythmias with sleep-disordered breathing. Am J Respir Crit Care Med.

[4] Epstein LJ, Kristo D, Strollo PJ, Friedman N, Malhotra A, Patil SP, Ramar K, Rogers R, Schwab RJ, Weaver EM, Weinstein MD (2009). Clinical guideline for the evaluation, management and long-term care of obstructive sleep apnea in adults. J Clin Sleep Med.

[5] Patel VN, Pierce BR, Bodapati RK, Brown DL, Ives DG, Stein PK (2017). Association of holter-derived heart rate variability parameters with the development of congestive heart failure in the cardiovascular health study. JACC Heart Fail.

[6] Diaz A, Bourassa MG, Guertin MC, Tardif JC (2005). Long-term prognostic value of resting heart rate in patients with suspected or proven coronary artery disease. Eur Heart J.

[7] Jouven X, Empana JP, Escolano S, Buyck JF, Tafflet M, Desnos M, Ducimetière P (2009). Relation of heart rate at rest and long-term (>20 years) death rate in initially healthy middle-aged men. Am J Cardiol.

[8] Guilleminault C, Winkle R, Connolly S, Melvin K, Tilkian A (1984). Cyclical variation of the heart rate in sleep apnoea syndrome. Lancet.

[9] Almazaydeh L, Elleithy K, Faezipour M (2012). Detection of obstructive sleep apnea through ECG signal features. Proceedings of the IEEE International Conference on Electro/Information Technology.

[10] Gardner M (2019). Development of a device for monitoring heart rate during Positive Airway Pressure therapy. Flinders University Theses Collections.

[11] Gardner M, Randhawa R, Malouf G, Reynolds KJ (2018). A modified mask for continuous cardiac monitoring during Positive Airway Pressure Therapy. Proceedings of the 40th Annual International Conference of the IEEE Engineering in Medicine and Biology Society (EMBC).

[12] Searle A, Kirkup L (2000). A direct comparison of wet, dry and insulating bioelectric recording electrodes. Physiol Meas.

[13] Allen J (2007). Photoplethysmography and its application in clinical physiological measurement. Physiol Meas.

[14] He DD, Winokur ES, Sodini CG (2015). An ear-worn vital signs monitor. IEEE Trans Biomed Eng.

[15] Alihanka J, Vaahtoranta K, Saarikivi I (1981). A new method for long-term monitoring of the ballistocardiogram, heart rate, and respiration. Am J Physiol Regul Integr Comp Physiol.

[16] Gardner M, Randhawa S, Malouf G, Reynolds KJ (2017). A wearable device for monitoring patients during PAP therapy. Proceedings of the IEEE Life Sciences Conference (LSC).

[17] Inan OT, Migeotte P, Park K, Etemadi M, Tavakolian K, Casanella R, Zanetti J, Tank J, Funtova I, Prisk GK, Di Rienzo M (2015). Ballistocardiography and seismocardiography: a review of recent advances. IEEE J Biomed Health Inform.

[18] Sadek I, Heng TT, Seet E, Abdulrazak B (2020). A new approach for detecting sleep apnea using a contactless bed sensor: comparison study. J Med Internet Res.

[19] Wang Z, Zhou X, Zhao W, Liu F, Ni H, Yu Z (2017). Assessing the severity of sleep apnea syndrome based on ballistocardiogram. PLoS One.

[20] Tadi MJ, Lehtonen E, Saraste A, Tuominen J, Koskinen J, Teräs M, Airaksinen J, Pänkäälä M, Koivisto T (2017). Gyrocardiography: a new non-invasive monitoring method for the assessment of cardiac mechanics and the estimation of hemodynamic variables. Sci Rep.

[21] Kim C, Carek AM, Mukkamala R, Inan OT, Hahn J (2015). Ballistocardiogram as proximal timing reference for pulse transit time measurement: potential for cuffless blood pressure monitoring. IEEE Trans Biomed Eng.

[22] Inan OT, Pouyan MB, Javaid AQ, Dowling S, Etemadi M, Dorier A, Heller JA, Bicen AO, Roy S, De Marco T, Klein L (2018). Novel wearable seismocardiography and machine learning algorithms can assess clinical status of heart failure patients. Circ Heart Fail.

[23] Rienzo MD, Vaini E, Lombardi P (2017). An algorithm for the beat-to-beat assessment of cardiac mechanics during sleep on earth and in microgravity from the seismocardiogram. Sci Rep.

[24] Hernandez J, Li Y, Rehg J, Picard R (2014). Bioglass: physiological parameter estimation using a head-mounted wearable device. Proceedings of the 4th International Conference on Wireless Mobile Communication and Healthcare - "Transforming healthcare through innovations in mobile and wireless technologies".

[25] Floris C, Solbiati S, Landreani F, Damato G, Lenzi B, Megale V, Caiani EG (2020). Feasibility of heart rate and respiratory rate estimation by inertial sensors embedded in a virtual reality headset. Sensors (Basel).

[26] Hernandez J, McDuff D, Picard R (2015). Biowatch: estimation of heart and breathing rates from wrist motions. Eur Union Digit Lib.

[27] Gardner M, Randhawa S, Reynolds KJ, Malouf G (2016). Estimation of heart rate during sleep measured from a gyroscope embedded in a CPAP mask. Proceedings of the IEEE EMBS Conference on Biomedical Engineering and Sciences (IECBES).

[28] Kushida CA, Chediak A, Berry RB, Brown LK, Gozal D, Iber C, Parthasarathy S, Quan SF, Rowley JA, Positive Airway Pressure Titration Task Force of the American Academy of Sleep Medicine (2008). Clinical guidelines for the manual titration of positive airway pressure in patients with obstructive sleep apnea. J Clin Sleep Med.

[29] Pan J, Tompkins WJ (1985). A real-time QRS detection algorithm. IEEE Trans Biomed Eng.

[30] Kim CS, Ober SL, McMurtry MS, Finegan BA, Inan OT, Mukkamala R, Hahn JO (2016). Ballistocardiogram: mechanism and potential for unobtrusive cardiovascular health monitoring. Sci Rep.

[31] Castanedo F (2013). A review of data fusion techniques. Sci World J.

[32] Li Q, Mark RG, Clifford GD (2008). Robust heart rate estimation from multiple asynchronous noisy sources using signal quality indices and a Kalman filter. Physiol Meas.

[33] Shen G, Ding R, Yang M, Han D, Zhang B (2020). An elastic manifold learning approach to beat-to-beat interval estimation with ballistocardiography signals. Adv Eng Inform.

[34] Lu H, Zhang H, Lin Z, Huat NS (2018). A novel deep learning based neural network for heartbeat detection in ballistocardiograph. Proceedings of the 40th Annual International Conference of the IEEE Engineering in Medicine and Biology Society (EMBC).

[35] Cathelain G, Rivet B, Achard S, Bergounioux J, Jouen F (2020). U-net neural network for heartbeat detection in ballistocardiography. Proceedings of the 42nd Annual International Conference of the IEEE Engineering in Medicine & Biology Society (EMBC).

